# Supplementary data for a focused review and meta-analysis of ^1^H-MRS studies on cerebral glutamate and GABA levels in high-risk of psychosis states

**DOI:** 10.1016/j.dib.2019.104920

**Published:** 2019-12-04

**Authors:** Christina Wenneberg, Birte Yding Glenthøj, Carsten Hjorthøj, Frederik Johan Buchardt Zingenberg, Louise Birkedal Glenthøj, Egill Rostrup, Brian Villumsen Broberg, Merete Nordentoft

**Affiliations:** a Copenhagen Research Center for Mental Health, CORE, Mental Health Center Copenhagen, Copenhagen University Hospital, Gentofte Hospitalsvej 15.4, 2900 Hellerup, Denmark; b Center for Neuropsychiatric Schizophrenia Research, CNSR, Center for Clinical Intervention and Neuropsychiatric Schizophrenia Research, CINS, Mental Health Centre Glostrup, University of Copenhagen, Ndr. Ringvej 29-67, 2600 Glostrup, Denmark; c University of Copenhagen, Department of Public Health, Section of Epidemiology, Øster Farimagsgade 5, Postboks 2099, 1014 Copenhagen K, Denmark

**Keywords:** Glutamate, GABA, UHR, Prodromal, High-risk, ^1^H-MRS

## Abstract

Data (attached) for a focused review and meta-analysis of cerebral levels of glutamate, Glx, and GABA levels assessed with ^1^H-MRS in high-risk of psychosis states was collected and stored at covidence.org and extracted to The Cochrane Collaboration Review Manager software package (RevMan Version 5.3) for meta-analytical purposes. Meta-analyses were performed with a random-effects, inverse-variance weighted model to calculate the pooled effect size. Heterogeneity was measured using the I^2^ value. Significance was assessed using two-sided 95% confidence intervals. Potential publication bias was assessed by visual inspection of funnel plots.

Supplementary to the co-submitted article are comprehensive meta-analyses of glutamate, Glx, and GABA, as well as the PRISMA flow diagram of included studies and a list of studies included in the review along with available measures and methodological variables.

The attached data offers an insight into the included studies and the specified metabolite values for each study and offers possible further investigation for other researchers, as well as an insight into the review and meta-analyses performed. The supplementary material also serves to support findings and interpretations in the main article.

**Table:** Specifications Table

Subject	Psychiatry and Mental Health
Specific subject area	Supplementary content to a focused review and meta-analysis of ^1^H-MRS studies on cerebral glutamate and GABA levels in high-risk of psychosis states
Type of data	Table Figure Diagram Plot
How data were acquired	Electronic searches in Medline and Embase for case-control studies without restrictions on language, year, or publication status. Search terms: (Ultra high risk or Genetic high risk or Clinical high risk or High risk) and (Glutamate* or GABA* or Neurotransmitter*) and (MRS or Spectroscopy or MR* or Magnetic resonance spectroscopy or ^1^H-MRS)). The Cochrane Collaboration Review Manager software package (RevMan Version 5.3) was used to perform meta-analyses.
Data format	Raw Analyzed Filtered
Parameters for data collection	A systematic review of all case-control studies that examined glutamate and/or GABA levels—measured with ^1^H-MRS—in individuals in high-risk states of psychosis states compared to healthy controls.
Description of data collection	The PRISMA group guidelines were followed. Searches were performed in Medline and Embase, and clinicaltrials.gov was searched for ongoing or unpublished studies. Risk of bias was assessed with the Newcastle-Ottawa scale for case-control studies. Two researchers performed the literature search (CW and FZ).
Data source location	Institution: Copenhagen Research Center for Mental Health, CORE, Mental Health Center Copenhagen, Copenhagen University Hospital City/Town/Region: Hellerup Country: Denmark Latitude and longitude (and GPS coordinates) for collected samples/data: 55.738580; 12.548360 (55°44′20.7″N 12°32′39.2″E)
Data accessibility	With the article
Related research article	Christina Wenneberg, Cerebral glutamate and GABA levels in high-risk of psychosis states: a focused review and meta-analysis of ^1^H-MRS studies, Schizophrenia Research Journal, Under review

**Table:** 

**Value of the Data** • The provided data are useful for gaining insight into the data underlying the review and meta-analysis. The supplementary data are useful for supporting points made in the main article. • Researchers showing further interest in the available literature on glutamatergic and GABAergic disturbances in high-risk for psychosis states, as well as those who read the article and wish to gain further insight into the underlying analyses, will benefit from the raw data and supplementary material. • Researchers wishing to perform additional analyses based on the available data, e.g., different subgroups will be able to extract and apply data to further research.

## 1 Data description

File I includes comprehensive meta-analyses for glutamate, Glx, and GABA as well as subgroup analyses according to the type of study (e.g., clinical or genetic high-risk; study performed on antipsychotic naïve participants or not).

**Fig. 1:**
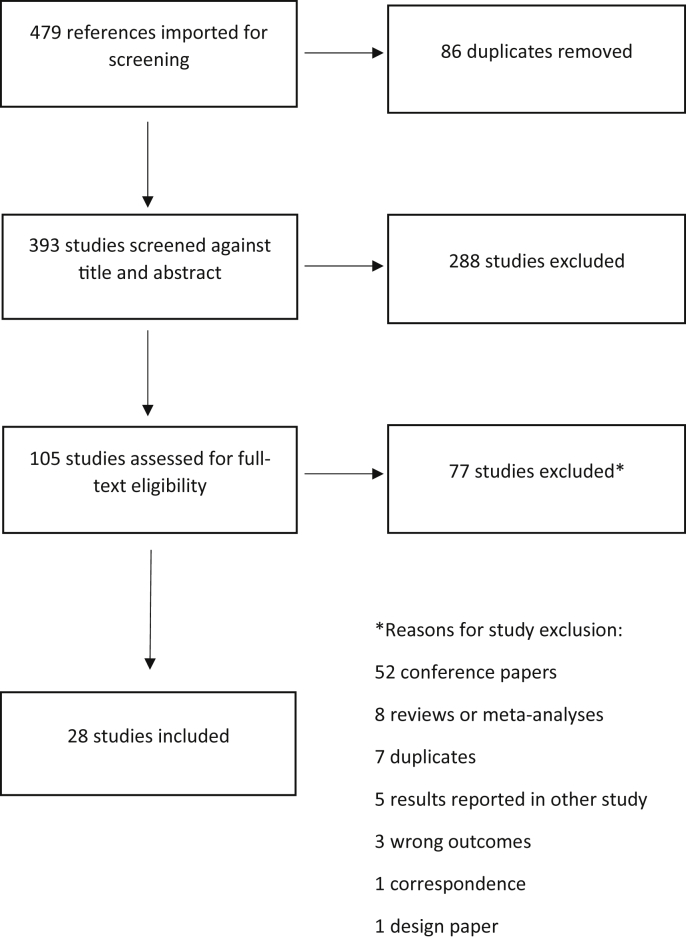
PRISMA flow diagram of literature search and study selection.

Fig. 1 depicts the PRISMA flow diagram of the included studies.

Table 1 lists the studies included in the review as well as all available measures and methodological variables.

**Table 1:** List of studies included in the review, available measures and methodological variables.

Study and Year	High-risk group	Region	Voxel size (cm)	Metabolite	Field strength (T)	Acquisition sequence	Echo time (ms)	Correction method	Mean (SD) in article
Block 2000	GHR	Left frontal lobe	4.0 × 3.0 × 2.5	Glx	1.5	PRESS	30	Cr scaling	Yes
		Basal ganglia	3.5 × 3.5 × 2.0						
Bloemen 2011	UHR	Left hippocampus	2 × 2 × 2	Glu	3	PRESS	36	Not stated	Yes
Byun 2009	UHR with MDD	ACC	2 × 2 × 2	Glx	1.5	PRESS	40	CSF	Yes
	UHR without MDD	Left DLPFC							
		Left thalamus							
Capizzano 2011	GHR (1rst degree relative)	ACC	8 cm^3^	Glx	3	PRESS	30	Cr scaling	Yes
	GHR (2nd degree relative)	Left hippocampus	4.5 cm^3^						
Da Silva 2018	CHR	MPFC	2 × 4 × 3	GABA	3	MEGA-PRESS	68	Not stated	Provided by author
de la Fuente-Sandoval 2011	UHR	Dorsal caudate	2 × 2 × 2	Glu, Glx	3	PRESS	35	CSF	Yes
		Cerebellum							
de la Fuente-Sandoval 2015	UHR	MPFC	3.0 × 2.5 × 2.5	Glx, GABA	3	MEGA-PRESS	68	CSF	Yes
		Dorsal caudate	4.5 × 2.5 × 2.0						
Egerton 2014	UHR	Left thalamus	1.5 × 2.0 × 2.0	Glu, Glx	3	PRESS	30	CSF	Yes
		ACC	2 × 2 × 2						
Grent-'t-Jong 2018	CHR	Right MOG	2 × 2 × 2	GABA, Glx	3	MEGA-PRESS	68	Cr scaling	Raw data available
Keshavan 2009	GHR	Caudate	4.5cm^3^	Glx	1.5	PRESS	30	CSF	Estimated
Liemburg 2016	UHR	Left LPFC	2 × 2 × 2	Glx	1.5	PRESS	144	CSF	Estimated
Lutkenhoff 2007	GHR	mesPFC	2 × 2 × 2	Glu	3	PRESS	30	CSF	Yes
		L-PFWM	2 × 2 × 2						
		Left hippocampus	1.5 × 1.5 × 1.5						
Marenco 2016	GHR	ACC	2 × 2 × 4.5	GABA	3	MEGA-PRESS	68	CSF	Provided by author
Menschikov 2016	UHR-S	ACC	3 × 3 × 3	GABA, Glx	3	MEGA-PRESS	68	Cr scaling	Provided by author
Modinos 2018	UHR	MPFC	4.0 × 3.5 × 2.0	GABA, Glu. Glx	3	MEGA-PRESS	68	Cr scaling	Corrected values provided by author
Natsubori 2013	UHR	MPFC	2 × 2 × 2	Glx	3	STEAM	15	CSF	Yes
Nenadic 2015	UHR	Right hippocampus	3 × 1 × 1	Glu	3	PRESS	30	CSF	Yes
		Left hippocampus							
Purdon 2007	GHR	MFC	2.5 × 2.5 × 2.5	Glu, Glx	3	STEAM	20	Cr scaling	Yes
Shakory 2018	CHR	Left hippocampus	3.0 × 1.4 × 1.0	Glu, Glx	3	PRESS	35	CSF	Estimated
			3.0 × 1.4 × 1.0						
Shendyapina 2015	CHR	DLPC	?	Glx	3	PRESS	35	Not stated	Not given
		Left thalamus							
		Right thalamus							
Stone 2009	ARMS	ACC	2 × 2 × 2	Glu, Gln, Glx	3	PRESS	30	CSF	Yes
		Left hippocampus	2.0 × 2.0 × 1.5						
		Left thalamus	1.5 × 2.0 × 2.0						
Tandon 2013	GHR	Thalamus	1.5 × 1.5 × 2.0	Glx	1.5	PRESS	30	CSF	Yes
		Caudate	1.5 × 1.5 × 2.0						
		ACC	1.5 × 1.5 × 2.0						
Thakkar 2017	GHR	Occipital cortex	4.0 × 2.4 × 2.5	GABA, Glu, Gln, Glx	7	MEGA-sLASER	36	Cr scaling	Yes
		Right striatum	4.0 × 2.4 × 2.5						
		Left striatum	4.0 × 2.4 × 2.5						
Tibbo 2004	GHR	Right MFC	2.5cm^3^	Glx	3	STEAM	20	Cr scaling	Yes
Valli 2011	ARMS	Medial temporal cortex	2.0 × 2.0 × 1.5	Glu	3	PRESS	30	CSF	Yes
		ACC	2 × 2 × 2						
		Thalamus	1.5 × 2.0 × 2.0						
Wang 2016	UHR	MPFC	3 × 3 × 3	GABA, Glx	3	MEGA-PRESS	68	CSF	Estimated
Wood 2010	UHR-P	Temporal lobe	?	Glx	3	PRESS	30	Not stated	Yes
	UHR-NP								
Yoo 2009	HGR (two relatives w. scz.)	ACC	2 × 2 × 2	Glx	1.5	PRESS	140	CSF	Yes
		DLPC	2.0 × 1.5 × 2.0						
		Thalamus	1.5 × 2.0 × 2.0						

Abbreviations: T, Tesla; GHR, genetic high risk; UHR, ultra-high risk; MDD, major depressive disorder; CHR, clinical high risk; ARMS, at-risk mental state; UHR-P, UHR with transition; UHR-NP, UHR without transition; HGR, high genetic risk; scz., schizophrenia; ACC, anterior cingulate cortex; DLPFC, dorsolateral prefrontal cortex; MPFC, medial prefrontal cortex; MOG, middle occipital gyrus; LPFC, lateral prefrontal cortex; MPFC, medial prefrontal cortex; PFWM, prefrontal white matter; Glx, combined measures of glutamate and glutamine; Glu, glutamate; GABA, gamma aminobutyric acid; PRESS, Point resolved spectroscopy; MEGA-PRESS, Meshcher–Garwood point resolved spectroscopy; STEAM, stimulated echo acquisition mode; Cr, creatine; CSF, cerebrospinal fluid.

**Fig. 2:**
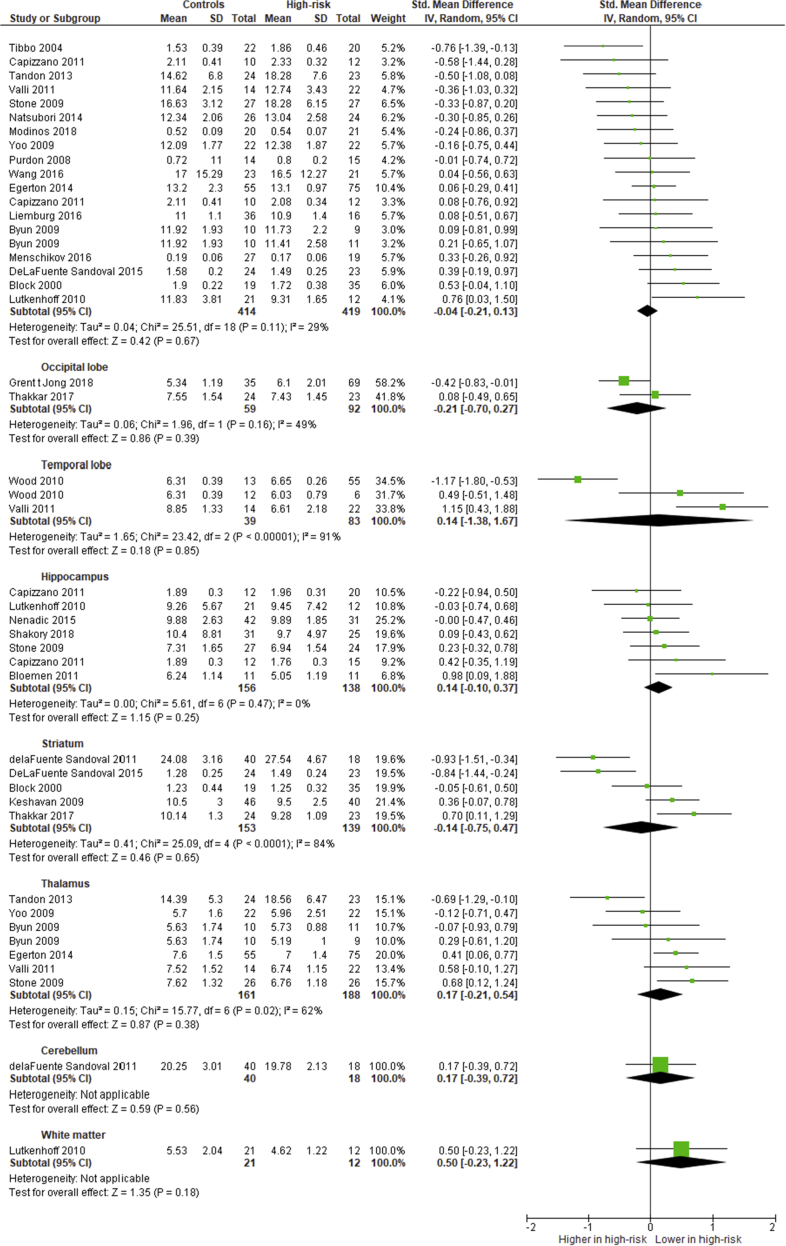
Meta-analysis of all glutamate and Glx studies included in the meta-analysis combined (including Glx measures for studies not reporting glutamate). Forest plot showing standardized mean differences for ^1^H-MRS glutamate studies in high-risk versus controls. Error bars represent 95% confidence intervals. Abbreviations: ^1^H-MRS, proton magnetic resonance spectroscopy.

Fig. 2 shows the meta-analysis and forest plot of all glutamate and Glx studies included in the review combined (including Glx measures for studies not reporting glutamate).

**Fig. 3:**
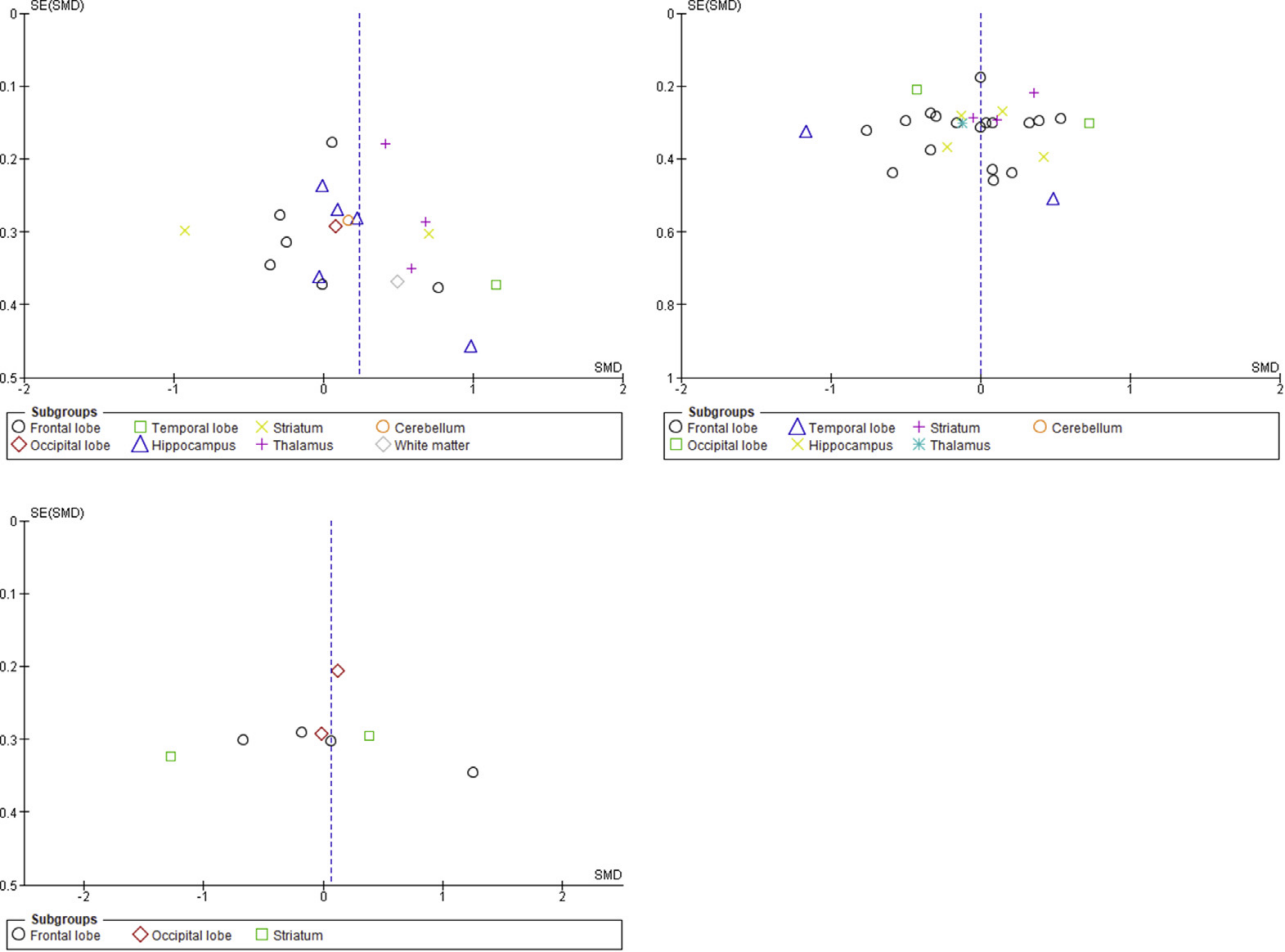
Funnel plot of comparison for studies included in the review. Top row left to right: Glutamate, Glx. Bottom row: GABA. Abbreviations: Glx, combined measures of glutamate and glutamine; GABA, gamma aminobutyric acid.

Fig. 3 depicts the funnel plot of comparisons for studies included in the review for glutamate, Glx, and GABA, respectively, to assess signs of asymmetry reflecting possible publication bias.

File II includes raw data of glutamate, Glx, and GABA levels for all included studies as well as author information and a range of demographical and clinical data extracted from the studies.

## 2 Experimental design, materials, and methods

The associated review and meta-analysis [1] was designed to establish whether there is any difference in baseline glutamate or GABA levels in individuals at high risk of developing psychosis (clinical or genetic high-risk states) compared to healthy controls as measured with ^1^H-MRS (levels of cerebral glutamate (glutamate, glutamine, or Glx) and/or GABA measured by applying a voxel of interest in a cerebral region) where HR and healthy controls are being compared.

High-risk states included clinical high risk as measured by clinical assessment tools (e.g., CAARMS, SIPS, or SOPS) as well as genetic high risk (having a relative with a psychotic disorder).

Embase and MEDLINE databases were searched for all relevant case/control studies without restrictions on language, year, or publication status. Studies fulfilling the search strategy were included search terms: (Ultra high risk or Genetic high risk or Clinical high risk or High risk) and (Glutamate* or GABA* or Neurotransmitter*) and (MRS or Spectroscopy or MR* or Magnetic resonance spectroscopy or ^1^H-MRS). If more than one scan was done in the same population, we only included the baseline values.

Reference lists of included studies and reviews were searched manually for additional studies, and clinicaltrials.gov were searched for ongoing or unpublished studies, and the responsible researchers were contacted and asked to share potential unpublished data. We received no unpublished data.

We excluded studies with no comparison group or with the wrong comparison group (e.g., not healthy controls such as patients with schizophrenia or first-episode psychosis) as well as studies with previously published results.

The first search was performed on March 10th, 2019, and the final search was performed on April 9th, 2019, where no new eligible studies were found.

Two researchers performed the literature search (CW and FZ). Titles and abstracts screening, as well as full-text screening, were done independently by the two above mentioned researches. Any disagreements between the two independent assessors were resolved through discussion, with CW having the final word. Data from each study were independently extracted from the included studies by CW and FZ. CW compared the extracted data and determined the consensus. We used covidence.org for importing studies, title and abstracts screening, full-text screening, and for extracting and storing the data. Primary data extracted were available metabolite levels of GABA, Glx, glutamate, or glutamine measured by ^1^H-MRS. Risk of bias assessment of included studies was performed using the Newcastle-Ottawa scale.

Data were subsequently exported to The Cochrane Collaboration Review Manager software package (RevMan Version 5.3) were meta-analyses were performed using a random-effects, inverse-weighted variance model to calculate the pooled effect size since studies were expected to display high heterogeneity based on different correction methods, varying regions of interest, and diverse high-risk groups. The study effect size was weighted according to sample size. Heterogeneity was measured using the I^2^ value, with higher percentages signifying higher variation across studies in the meta-analysis. Significance was assessed using two-sided 95% confidence intervals.

Meta-analyses were performed separately for mean values of GABA, glutamate, and Glx (only two studies reported glutamine levels; hence, no meta-analysis was performed for this metabolite). If means or standard deviations were not published or only reported in figures, authors were contacted for this information. If we did not receive a response, means and SDs were estimated from represented figures using WebPlotDigitizer or—if not shown in figures—not included in the meta-analysis (Table 1 provides more detail on this). Potential publication bias was assessed by visual inspection of funnel plots for studies on glutamate, Glx, and GABA, respectively (Fig. 3).

Each metabolite results was sorted into relevant subdomains of the brain ((1)frontal lobe, voxels placed in the anterior cingulate cortex (ACC), the medial prefrontal cortex (MPFC), or the dorsolateral prefrontal cortex (DLPFC); (2)the occipital lobe; (3)the temporal lobe; (4)hippocampus; (5)striatum, including basal ganglia and caudate; (6)thalamus; (7)cerebellum; and (8)white matter). Measures were not available for all regions for all metabolites. Each study was only represented once in each subdomain. For studies reporting subgroups of high-risk states with a shared control group, measures were treated as separate data sets, and the number of healthy controls was divided by the number of subgroups (always two). In studies where bilateral measures were provided, measures from the left side were included in the analyses.

When the number of studies allowed (more than two studies available), subgroup analyses were performed based on high-risk status, treatment with antipsychotic medication, and location of the voxel of interest in the frontal lobe.

## Conflict of Interest

CW has received a Ph.D. grant from The Research Foundation of the Mental Health Services in the Capital Region of Denmark.

The authors declare that they have no known competing financial interests or personal relationships that could have appeared to influence the work reported in this paper.
