# Supplementary material for: Supplementary data for a focused review and meta-analysis of 1H-MRS studies on cerebral glutamate and GABA levels in high-risk of psychosis states
Source: Data Brief. 2019 Dec 4;28:104920. doi: 10.1016/j.dib.2019.104920 (PMC6920491; doi:10.1016/j.dib.2019.104920)
Supplement: Multimedia component 2 [file mmc2.docx]

## Supplementary material

### Comprehensive meta-analyses

#### Glutamate

A comprehensive meta-analysis for all studies reporting glutamate [1-11] including only one measure per study (11 data sets, 279 HR, 299 controls). We included the data set with the highest weight in the meta-analysis for studies with more than one voxel of interest. The meta-analysis returned no significant difference (SMD = 0.01 (95% CI [-0.16, 0.18], P = 0.92) with I² = 0% (P=0.62)).

Subgroup analyses were done separating data sets into (i) clinical [1, 3-5, 7, 8, 10, 11] or genetic HR [2, 6, 9]; or (ii) AP naïve studies [2, 4, 6, 7, 9, 11]. None of these subgroup analyses returned any significant difference.

#### Glx

For all studies reporting Glx [1, 2, 4, 5, 7, 9, 10, 12-25] (including only the highest weighing data set for multi-voxel studies) the difference is non-significant (24 data sets, 634 HR, 584 HC, SMD = -0.08 (95% CI [-0.24, 0.09], P = 0.35) with I² = 45% (P = 0.009)). Subgroup analyses were done separating data sets into (i) clinical [1, 4, 5, 7, 10, 13, 15, 16, 18-20, 23, 24] or genetic HR [2, 9, 12, 14, 17, 21, 22, 25]; or (ii) AP naïve studies [2, 4, 7, 9, 12, 14, 15, 17, 18, 21-25]. None of these subgroup analyses returned any significant difference.

#### GABA

A meta-analysis for all studies reporting GABA [2, 7, 15, 16, 19, 23, 26, 27] (including only the highest weighing data set for multi-voxel studies) was non-significant (8 data sets, 243 HR, 356 HC, SMD = 0.30 (95% CI [-0.13, 0.72], P = 0.17) with I² = 80% (P < 0.0001)). Subgroup analyses with exclusion of the one study reporting findings on a genetic HR group [26] and two studies with a “mixed” AP group [19, 27] respectively did not result in significant findings.

[1] Stone JM, Day F, Tsagaraki H, Valli I, McLean MA, Lythgoe DJ, et al. Glutamate dysfunction in people with prodromal symptoms of psychosis: relationship to gray matter volume. Biol Psychiatry 2009;66(6):533-9.

[2] Thakkar KN, Rosler L, Wijnen JP, Boer VO, Klomp DW, Cahn W, et al. 7T Proton Magnetic Resonance Spectroscopy of Gamma-Aminobutyric Acid, Glutamate, and Glutamine Reveals Altered Concentrations in Patients With Schizophrenia and Healthy Siblings. Biological psychiatry 2017;81(6):525-35.

[3] Bloemen OJ, Gleich T, de Koning MB, da Silva AF, de HL, Linszen DH, et al. Hippocampal glutamate levels and striatal dopamine D(2/3) receptor occupancy in subjects at ultra high risk of psychosis. Biol Psychiatry 2011;70(1):e1-e2.

[4] de la Fuente-Sandoval C, Leon-Ortiz P, Favila R, Stephano S, Mamo D, Ramirez-Bermudez J, et al. Higher levels of glutamate in the associative-striatum of subjects with prodromal symptoms of schizophrenia and patients with first-episode psychosis. Neuropsychopharmacology 2011;36(9):1781-91.

[5] Egerton A, Stone JM, Chaddock CA, Barker GJ, Bonoldi I, Howard RM, et al. Relationship between brain glutamate levels and clinical outcome in individuals at ultra high risk of psychosis. Neuropsychopharmacology 2014;39(12):2891-9.

[6] Lutkenhoff ES, van Erp TG, Thomas MA, Therman S, Manninen M, Huttunen MO, et al. Proton MRS in twin pairs discordant for schizophrenia. Molecular psychiatry 2010;15(3):308-18.

[7] Modinos G, Simsek F, Horder J, Bossong M, Bonoldi I, Azis M, et al. Cortical GABA in subjects at ultra-high risk of psychosis: Relationship to negative prodromal symptoms. International Journal of Neuropsychopharmacology 2018;21(2):114-9.

[8] Nenadic I, Maitra R, Basu S, Dietzek M, Schonfeld N, Lorenz C, et al. Associations of hippocampal metabolism and regional brain grey matter in neuroleptic-naive ultra-high-risk subjects and first-episode schizophrenia. European Neuropsychopharmacology 2015;25(10):1661-8.

[9] Purdon SE, Valiakalayil A, Hanstock CC, Seres P, Tibbo P. Elevated 3T proton MRS glutamate levels associated with poor Continuous Performance Test (CPT-0X) scores and genetic risk for schizophrenia. Schizophrenia research 2008;99(1-3):218-24.

[10] Shakory S, Watts JJ, Hafizi S, Da Silva T, Khan S, Kiang M, et al. Hippocampal glutamate metabolites and glial activation in clinical high risk and first episode psychosis. Neuropsychopharmacology 2018.

[11] Valli I, Stone JM, Mechelli A, Bhattacharyya S, Raffin M, Poli PF, et al. Altered medial temporal activation related to local glutamate in subjects with prodromal signs of psychosis. Schizophrenia research 2010;117 (2-3):533.

[12] Block W, Bayer TA, Tepest R, Traber F, Rietschel M, Muller DJ, et al. Decreased frontal lobe ratio of N-acetyl aspartate to choline in familial schizophrenia: a proton magnetic resonance spectroscopy study. Neurosci Lett 2000;289(2):147-51.

[13] Byun MS, Choi JS, Yoo SY, Kang DH, Choi CH, Jang DP, et al. Depressive Symptoms and Brain Metabolite Alterations in Subjects at Ultra-high Risk for Psychosis: A Preliminary Study. Psychiatry Investig 2009;6(4):264-71.

[14] Capizzano AA, Toscano JL, Ho BC. Magnetic resonance spectroscopy of limbic structures displays metabolite differences in young unaffected relatives of schizophrenia probands. Schizophrenia research 2011;131(1-3):4-10.

[15] De La Fuente-Sandoval C, Reyes-Madrigal F, Mao X, Leon-Ortiz P, Rodriguez-Mayoral O, Solis-Vivanco R, et al. Cortico-striatal GABAergic and glutamatergic dysregulations in subjects at ultra-high risk for psychosis investigated with proton magnetic resonance spectroscopy. International Journal of Neuropsychopharmacology 2015;19(3):1-10.

[16] Grent-'t-Jong T, Gross J, Goense J, Wibral M, Gajwani R, Gumley AI, et al. Resting-state gamma-band power alterations in schizophrenia reveal E/I-balance abnormalities across illness-stages. eLife 2018;7.

[17] Keshavan MS, Dick RM, Diwadkar VA, Montrose DM, Prasad KM, Stanley JA. Striatal metabolic alterations in non-psychotic adolescent offspring at risk for schizophrenia: a (1)H spectroscopy study. Schizophrenia research 2009;115(1):88-93.

[18] Liemburg E, Sibeijn-Kuiper A, Bais L, Pijnenborg G, Knegtering H, van der Velde J, et al. Prefrontal NAA and Glx Levels in Different Stages of Psychotic Disorders: a 3T 1H-MRS Study. Scientific reports 2016;6:21873.

[19] Menschikov PE, Semenova NA, Ublinskiy MV, Akhadov TA, Keshishyan RA, Lebedeva IS, et al. (1)H-MRS and MEGA-PRESS pulse sequence in the study of balance of inhibitory and excitatory neurotransmitters in the human brain of ultra-high risk of schizophrenia patients. Doklady Biochemistry and biophysics 2016;468(1):168-72.

[20] Natsubori T, Inoue H, Abe O, Takano Y, Iwashiro N, Aoki Y, et al. Reduced Frontal Glutamate + Glutamine and N-Acetylaspartate Levels in Patients With Chronic Schizophrenia but not in Those at Clinical High Risk for Psychosis or With First-Episode Schizophrenia. Schizophr Bull 2013.

[21] Tandon N, Bolo NR, Sanghavi K, Mathew IT, Francis AN, Stanley JA, et al. Brain metabolite alterations in young adults at familial high risk for schizophrenia using proton magnetic resonance spectroscopy. Schizophrenia research 2013;148(1-3):59-66.

[22] Tibbo P, Hanstock C, Valiakalayil A, Allen P. 3-T proton MRS investigation of glutamate and glutamine in adolescents at high genetic risk for schizophrenia. The American journal of psychiatry 2004;161(6):1116-8.

[23] Wang J, Tang Y, Zhang T, Cui H, Xu L, Zeng B, et al. Reduced gamma-Aminobutyric Acid and Glutamate+Glutamine Levels in Drug-Naive Patients with First-Episode Schizophrenia but Not in Those at Ultrahigh Risk. Neural plasticity 2016;2016:3915703.

[24] Wood SJ, Kennedy D, Phillips LJ, Seal ML, Y++cel M, Nelson B, et al. Hippocampal pathology in individuals at ultra-high risk for psychosis: A multi-modal magnetic resonance study. NeuroImage 2010;52(1):62-8.

[25] Yoo SY, Yeon S, Choi CH, Kang DH, Lee JM, Shin NY, et al. Proton magnetic resonance spectroscopy in subjects with high genetic risk of schizophrenia: investigation of anterior cingulate, dorsolateral prefrontal cortex and thalamus. Schizophrenia research 2009;111(1-3):86-93.

[26] Marenco S, Meyer C, Kuo S, van der Veen JW, Shen J, DeJong K, et al. Prefrontal GABA Levels Measured With Magnetic Resonance Spectroscopy in Patients With Psychosis and Unaffected Siblings. The American journal of psychiatry 2016;173(5):527-34.

[27] Da Silva T, Hafizi S, Rusjan PM, Houle S, Wilson AA, Prce I, et al. GABA levels and TSPO expression in people at clinical high risk for psychosis and healthy volunteers: a PET-MRS study. Journal of psychiatry & neuroscience : JPN 2019;44(2):111-9.
